# Retraction: Eldefrawy, M.H.; Khan, M.K.; Alghathbar, K.; Tolba, A.S.; Kim, K.J. Authenticated Key Agreement with Rekeying for Secured Body Sensor Networks. *Sensors* 2011, *11*, 5835–5849.

**DOI:** 10.3390/s110807992

**Published:** 2011-08-15

**Authors:** Shu-Kun Lin

**Affiliations:** MDPI AG, Postfach, CH-4005 Basel, Switzerland; Office: Kandererstrasse 25, 4057 Basel, Switzerland; E-Mail: lin@mdpi.com

It has been brought to our attention by a reader of *Sensors* that substantial portions of this article [1] have been copied from earlier publications without credit. After confirming this plagiarism with the authors, we have determined that indeed this manuscript clearly violates our policy on originality of all material submitted for publication and the generally accepted ethics of scientific publication. Consequently, the Editorial Team and Publisher have determined that it should be retracted. We apologize for any inconvenience this may cause.
